# Deciphering the pathological significance of self‐propagating Aβ strains in different animal models

**DOI:** 10.1002/alz.094998

**Published:** 2025-01-09

**Authors:** Rodrigo Morales

**Affiliations:** ^1^ The University of Texas Health Science Center at Houston, Houston, TX USA

## Abstract

**Background:**

Alzheimer’s disease (AD) is an heterogenous disorder characterized by the accumulation of amyloid‐beta (Aβ) and tau. One possible explanation for the clinical and pathological variation in AD lies in the presence of distinct conformational strains of Aβ. Numerous studies provide compelling evidence for the existence of such strains as well as their ability to template their conformations in a prion‐like manner. However, the interaction of such Aβ strains with different hosts has yet to be thoroughly examined. Here, we examined the host‐seed interaction of different human brain‐derived and synthetic misfolded Aβ strains in two different mouse models of amyloid pathology. We specifically explored the potential differences in amyloid propagation and pathological manifestations considering Aβ strains and animal models as variables.

**Method:**

50‐day‐old Tg2576 mice were intra‐cerebrally challenged with mouse or human brain‐derived Aβ seeds, or synthetic Aβ strains. Age‐matched APP/PS1 mice were intra‐cerebrally challenged with the biological Aβ seeds as well. Cerebral pathology was assessed in 300‐day‐old Tg2576 mice, and in 180‐day‐old APP/PS1 mice.

**Result:**

Two structurally defined synthetic misfolded Aβ strains induced remarkably different pathological features in Tg2576, including different rates of aggregation, tropism to specific brain regions, and differential recruitment of Aβ40/Aβ42 peptides. Moreover, the administration of human brain homogenates derived from AD patients, displaying unique patterns of amyloidosis, seeded distinct phenotypes in challenged APP/PS1 mice, as evaluated by their seeding activity, vascular pathology, and reactivity to thioflavin‐S. Finally, we observed that the same human brain homogenate induced a different cerebral Aβ pathology when inoculated into Tg2576 and APP/PS1 mice.

**Conclusion:**

Our findings support the concept of Aβ strains and demonstrate that they may drive different clinical and pathological outcomes in humans. Our work highlights the contribution of the host in pathological characteristics. Future research could lead to successful personalized diagnostic approaches and treatments.

